# *In vitro* evaluation of *Penicillium chrysogenum* Snef1216 against *Meloidogyne incognita* (root-knot nematode)

**DOI:** 10.1038/s41598-020-65262-z

**Published:** 2020-05-20

**Authors:** Aatika Sikandar, Mengyue Zhang, Yuanyuan Wang, Xiaofeng Zhu, Xiaoyu Liu, Haiyan Fan, Yuanhu Xuan, Lijie Chen, Yuxi Duan

**Affiliations:** 1 Nematology Institute of Northern China, Shenyang Agricultural University, Shenyang, 110866 P.R. China; 2 College of Biosciences and Biotechnology, Shenyang Agricultural University, Shenyang, 110866 P.R. China; 3 College of Science, Shenyang Agricultural University, Shenyang, 110866 P.R. China; 4 College of Plant Protection, Shenyang Agricultural University, Shenyang, 110866 P.R. China

**Keywords:** Microbiology, Zoology

## Abstract

Root-knot nematode (*Meloidogyne incognita*) is chief plant parasitic nematode of various crops globally. Meanwhile, the negative side effects on human health and environmental concerns associated with haphazard uses of chemical nematicides. Hence, the search for a safe and effective approach is more relevant. The present study was aimed to evaluate the nematicidal potential of Snef1216 (*Penicillium chrysogenum*) against *M. incognita* at different concentrations (5%, 10%, 25%, 50%, 75% and 100%) and with the nutritious medium. The egg hatching inhibition and mortality of second stage juveniles of *M. incognita* were assessed after 6, 12, 24, 48 and 72 h exposure. Results revealed that egg hatching inhibition and percent mortality of *M. incognita* increased with increasing concentration and exposure time. The highest mortality of juveniles was recorded at 100% conc. i.e., 24.20%, 36%, 66%, 78% and 97.8% at 6, 12, 24, 48 and 72 h, respectively. The highest ovicidal activity was recorded at 100% concentration with 5.20% of eggs hatching. The outcome suggested that Snef1216 (*P. chrysogenum*) resulted in the lowest LC_50_ value was recorded as 3718.496 at 6 h exposure period followed by 10479.87, 11186.682, 14838.58 and 24001.430 at 72, 12, 48 and 24 h respectively via ovicidal assay. Whereas, in the larvicidal assay, the lowest LC_50_ value demonstrated at 72 h being 17.628% exposure period followed by 28.345, 50.490, 215.710 and 482.595% at 48, 24, 12 and 6 h respectively. It is concluded that Snef1216 has potential being used as a biocontrol agent against *M. incognita* and can serve as a source of a novel nematicidal agent of fungal origin.

## Introduction

Plant parasitic nematodes cause severe losses to the variety of crops by affecting their growth and yield^1^. They can easily damage the crops not only by feeding but also develop the association with other organisms possess a risk to agriculture globally with an estimated annual loss reached to 100–150 billion US dollars, more than half the losses only occurred by root-knot nematodes^2^. Root-knot nematodes (RKNs) are sedentary obligate endoparasitic in nature and are one of the major restraints in the production of economically important crops^3,4^.

*Meloidogyne* belongs to the one of the most damaging genus of root-knot nematodes owing to their polyphagous nature^5,6^. However, four common species of this genus, i.e. *Meloidogyne arenaria*, *M. incognita*, *M. hapla* and *M. javanica* have been reported as hazardous^7^. Among these species, *M. incognita* is the most damaging due to its extensive host range, high reproduction rate, the capability to produce complicated diseases with other pathogens and short generation time^8^. Plants are infested by root-knot nematodes showed the galls or knots on roots^9^ which disrupt the uptake of water and mineral, resulting in wilting of the plant, chlorosis, reduced tillering, excessive root branching, immature fruit drop, drying of leaf and stunted growth^10–12^. Its infection has the ability to reduce chlorophyll contents and alter numerous biochemical such as amino acids and organic acids^13^. At present, *M. incognita* can be controlled successfully by using chemical nematicides but they are hazardous for human health, other non-target organisms and environment^14,15^. Keeping in view the above described concerns; there is an urgent need to introduce long term integrative strategies and development of ecofriendly nematicides^16–18^.

Biological control is a safe way to control pests and pathogens. However, antagonists and nematophagous microorganisms are the best potential substitutes for chemical nematicides. Few nematophagous bacteria and fungi are commercially available to control plant parasitic nematodes^19–22^. Among biocontrol agents fungi have complex strategies for capturing the nematodes by sticky branches, non-constricting and constricting rings, killing by producing toxic substances, and digesting by colonizing their reproductive structures^23–25^.

Fungi belong to genera *Penicillium, Fusarium, Paecilomyces, Trichoderma, Purpurocillium , Clonostachys, Chaetomium, Phyllosticta, Isaria, Arthrobotrys, Verticillium and Acremonium* have been known as nematophagous or antagonistic to nematodes^26–28^. Over the recent years, the biocontrol effect against different pests and pathogens in the presence of Penicillium chrysogenum  has been reported in a variety of plants and pathogens, thus providing evidence that it can be used to control nematode infection, but the available data concerning with the application of *P. chrysogenum* to control nematode is little so far^29–32^. Murali, *et al*.^33^ described the importance of *P. chrysogenum* that it efficiently promotes growth, induced defence-related genes and produced disease resistance against downy mildew in pearl millet. Dry mycelium of *P. chrysogenum* reduced the root galls to protect tomato and cucumber plants against *Meloidogyne javanica* and enhanced plant growth^34^. Siddiqui and Akhtar^26^ demonstrated that *P. chrysogenum* used alone or with the combination of Arbuscular mycorrhizal fungi AMF, Plant growth-promoting rhizobacteria PGPRS and *Aspergillus niger* could reduce nematode infection. Thus keeping in mind the biocontrol potential of *P. chrysogenum*, the current study was planned to evaluate the ovicidal and larvicidal efficacy of fungus fermentation Snef 1216 (*P. chrysogenum*) against *M. incognita* under laboratory conditions at Nematology Institute of Northern China (NINC) of Shenyang Agricultural University, P.R. China.

## Results

### Ovicidal assay

Nematicidal potential of Snef1216 was assessed on egg hatching inhibition for *M. incognita* (Table 1). Matured egg masses were selected and also insured eggs had the same embryonic stage. Results showed that eggs hatched in medium and distilled water were higher than those hatched by Snef1216. As mean hatching was observed in the medium as found to be statistically similar to that observed in distilled water, it showed that the medium itself did not show ovistatic or ovicidal property. The results revealed significant differences among the concentration and exposure period. The results of the tested fermentation concentrations recommended that all concentrations had antagonistic effects on egg hatching. On increasing exposure period up to 72 hours resulted in increased egg hatching. Whereas, increasing dilution of fermentation, the cumulative hatching was increased. Maximum inhibition in egg hatching was attained by 100% conc. of Snef1216. The highest percentage hatching of *M. incognita* was 86.8% in distilled water (Control) followed by nutritious medium and at 5% concentration of fermentation 83.2 and 33.8% respectively at 72 h exposure. The highest ovicidal activities were recorded at 100% conc. of fermentation with approximately 5.20% eggs hatched. Egg hatching inhibition of *M. incognita* increased with increasing concentration and exposure time. Unhatched eggs were transferred into distilled water to check their performance in the absence of Snef1216. However, it showed the strong ovicidal potential by exhibiting the egg hatching.

**Table 1:** Effect of Snef1216 on eggs hatching of *M. incognita*.

Treatments (conc.)	6 h	12 h	24 h	48 h	72 h
100%	0 ± 0^b^	0 ± 0 ^f^	0 ± 0 ^g^	0 ± 0 ^f^	5.20 ± 1.64 ^h^
75%	0 ± 0^b^	0 ± 0 ^f^	0 ± 0 ^g^	0 ± 0 ^f^	7.4 ± 2.07^gh^
50%	0 ± 0^b^	0 ± 0 ^f^	3.0 ± 0.71 ^f^	6.40 ± 2.07^e^	10.0 ± 1.58 ^f^
25%	0 ± 0^b^	3.60 ± 1.14^e^	7.0 ± 1.58^e^	12.2 ± 1.30^d^	15.2 ± 2.49^e^
10%	0 ± 0^b^	6.40 ± 2.07^d^	13.4 ± 2.07^d^	21.6 ± 2.41^c^	24.6 ± 2.70^d^
5%	0 ± 0^b^	9.00 ± 1.58^c^	28.2 ± 2.39^c^	32.8 ± 2.39^b^	33.8 ± 2.39^c^
N. medium	7.60 ± 1 0.95^a^	29.8 ± 1.92^b^	56.8 ± 2.39^b^	69.0 ± 2.92^a^	83.2 ± 1.79^b^
CK (water)	8.60 ± 1.67^a^	33.2 ± 2.28^a^	59.2 ± 2.78^a^	70.6 ± 2.30^a^	86.8 ± 2.78^a^
**ANOVA test**					
S.S	494.58	6423.50	21262.30	29138.58	38795.58
M.S	70.66	917.64	3037.47	4162.65	5542.23
df	7	7	7	7	7
*F*	85.64	431.83	920.45	1063.94	1119.64
*P*	0.000	0.000	0.000	0.000	0.000

Data presents the mean ± standard deviation. The same letter within columns are significantly similar according to Duncan’s multiple range test (*P* > 0.05). Whereas; S.S (Sum of square); M.S (Mean square); df (Degree of freedom); *F* (F-value); *P* (significant value).

The data presented in (Table 2) revealed the ovicidal activity of *P. chrysogenum* against *M. incognita*. The hatching percentage was directly related to the concentration of fermentation and exposure time. Results displayed highly significant (P < 0.001) model fitness with (*F* = 533.28, 16594.74, 243.03, 0.72 and 1525.50) corrected model, intercept, concentration, replications and time period. The interaction/correlation between hatching versus concentration of fermentation and treatment recorded significant correlation with (P < 0.05), whereas the concentration of fermentation and exposure time also recorded highly significant (P < 0.001) positive correlation being (F = 18.67). The two-way analysis of variance provides information regarding the interaction between hatching percentage of *M. incognita* versus replication and exposure time recorded significant results (F = 1.30). Interaction/correlation between (concentration × replication × time) to total variance was strongly trait specific recorded significant hatching (P < 0.05).

**Table 2:** Analysis of variance of the effect of hatching (%) versus concentration, replication and time.

Source	S.S	M.S	df	F	Sig.
C.M	93012.69	1576.49	59	533.28	0.00
I	49057.22	49057.22	1	16594.74	0.00
C	5029.08	718.44	7	243.03	0.00
r	8.48	2.12	4	0.72	0.58
T	18038.61	4509.65	4	1525.49	0.00
C × r	0.00	.	0	.	.
C × T	1545.18	55.19	28	18.68	0.00
r × T	61.25	3.83	16	1.30	0.21
C × r × T	0.00	.	0	.	.

Whereas: S.S (Sum of square); M.S (Mean square); df (Degree of freedom); C.M (Corrected model); I (Intercept); C (Concentration); r (Replication); T (Time); C × r (Concentration × Replication); C × T (Concentration × Time); r × T (replication × Time); C × r × T (Concentration × replication × Time).

### Larvicidal assay

**Figure 1:**
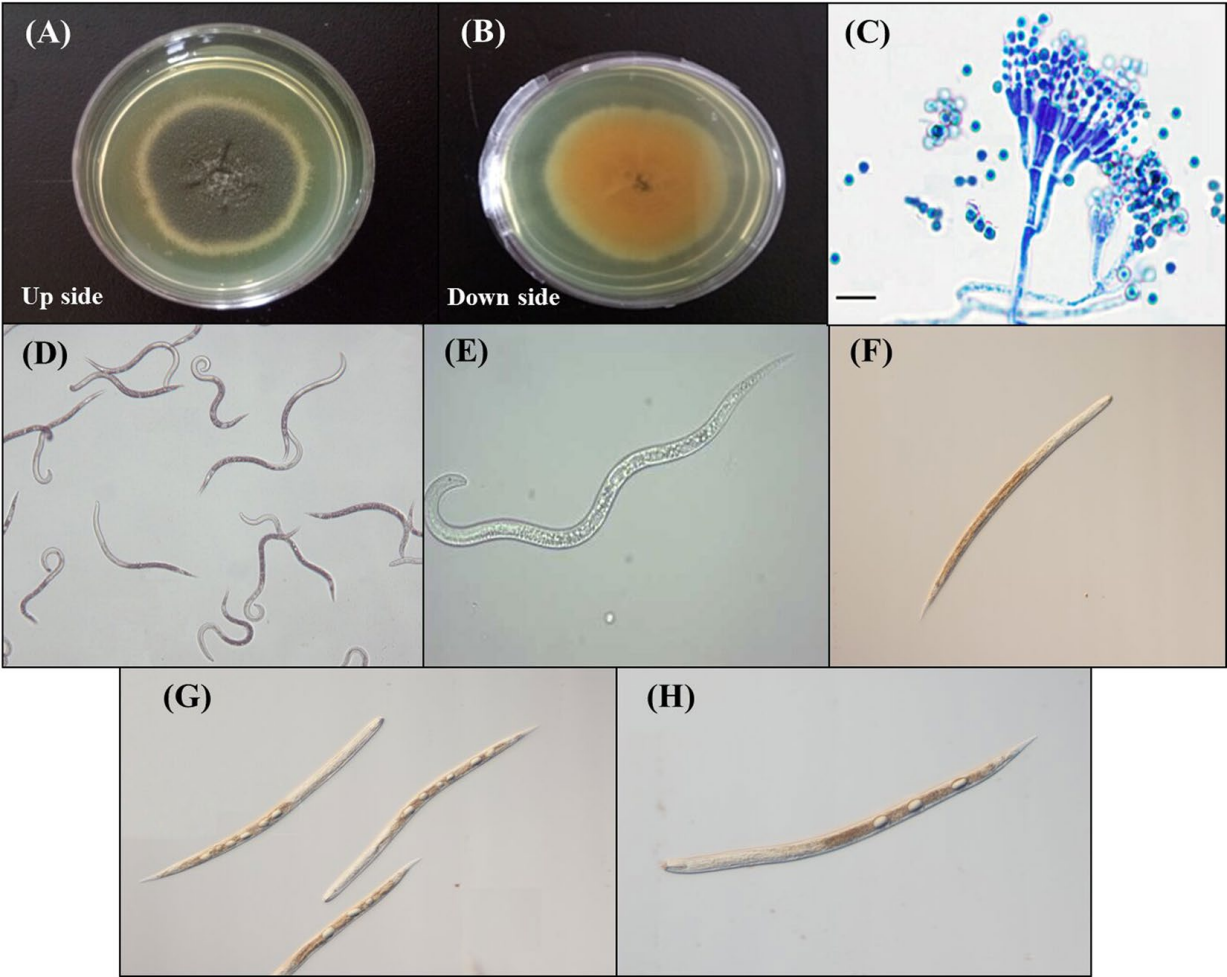
Pictorial representation of *P. chrysogenum* Snef1216 and its influence upon the entire body of second-stage juveniles (J2s). Whereas; (**A** and **B**) Pure fungal colony of *P. chrysogenum* Snef1216, (**C**) Microscopic image of *P. chrysogenum*, (**D** and **E**): Living juveniles, (**F**) Natural death of juvenile, (**G** and **H**): Juveniles death due to *P. chrysogenum* Snef1216.

It is apparent from the perusal of the data presented in Table 3 that mean percent mortality in the fermentation of the Snef1216 (*P. chrysogenum*) differed significantly from that in the medium and it is concluded that medium itself had no nematostatic or nematicidal effect as the mean mortality observed in the medium was statistically similar to distilled water. Data displayed in Table 3 exposed that all concentrations of fermentation showed the nematicidal effect on *M. incognita*. The activity of Snef1216 was the highest, with J2s mortality 24.2%, 36%, 66%, 78% and 97.8% for 6, 12, 24, 48 and 72 h, respectively at 100% concentration. A progressive increase in the concentrations of the fermentation resulted in increased mortality of J2s. Second-stage juveniles gradually reduced their movement within 48 h and mostly were immovable after 72 h (Fig. 1). After exposure to Snef1216, immovable second-stage juveniles were transferred into distilled water but they showed no resumption of mortality.

**Table 3:** Effect of Snef1216 against *M. incognita* juveniles.

Treatments (Conc.)	6 h	12 h	24 h	48 h	72 h
100%	24.2 ± 1.92^a^	36.0 ± 2.74^a^	66.0 ± 2.74^a^	78.0 ± 2.23^a^	97.8 ± 2.28^a^
75%	11.0 ± 1.58^b^	26.8 ± 1.92^b^	53.8 ± 2.39^b^	68.2 ± 1.92^b^	86.2 ± 2.78^b^
50%	8.8 ± 1.92^c^	22.6 ± 2.70^c^	47.0 ± 2.92^c^	58.2 ± 2.28^c^	70.0 ± 2.24^c^
25%	2.0 ± 1.58^d^	10.2 ± 1.92^d^	37.2 ± 2.86^d^	46.4 ± 2.07^d^	56.8 ± 2.86^d^
10%	0 ± 0^e^	5.0 ± 1.58^e^	29.6 ± 2.07^e^	35.0 ± 2.65^e^	39.6 ± 2.70^e^
5%	0 ± 0^e^	0.8 ± 0.84 ^f^	26.8 ± 1.92^e^	30.8 ± 2.39 ^f^	32.6 ± 1.82 ^f^
N. medium	0 ± 0^e^	0.4 ± 0.55 ^f^	4.6 ± 1.95 ^f^	10.0 ± 2.74 ^g^	13.4 ± 2.51 ^g^
CK (water)	0 ± 0^e^	0 ± 0 ^g^	1.0 ± 0.71 ^g^	5.40 ± 2.07 ^h^	7.6 ± 1.81 ^h^
**ANOVA test**					
S.S	2617.90	6797.18	18076.70	24001.20	37938.80
M.S	373.99	971.03	2582.39	3428.74	5419.83
df	7	7	7	7	7
*F*	241.28	302.27	489.55	642.39	936.47
*P*	0.000	0.000	0.000	0.000	0.000

Data presents the mean ± standard deviation. The same letter within columns are significantly similar according to Duncan’s multiple range test (*P* > 0.05). Whereas; S.S (Sum of square); M.S (Mean square); df (Degree of freedom); *F* (F-value); *P* (significant value).

The data presented in (Table 4) revealed the larvicidal activity of *P. chrysogenum* against *M. incognita*. The percent mortality was directly related to the concentration of fermentation and exposure time. Outcomes displayed highly significant (P < 0.001) model fitness with (*F* = 585.09, 34637.48, 154.58, 0.819 and 3075.95) corrected model, intercept, concentration, replication and time period. The interaction/correlation between mortality versus concentration of fermentation and replication recorded a highly significant correlation with (P < 0.05), whereas the concentration of fermentation and exposure time also recorded highly significant (P < 0.001) positive correlation with (F = 11.58). The two-way analysis of variance provides information regarding the interaction between mortality (%) of *M. incognita* versus replication and exposure time recorded significant results (F = 0.97). Interaction/correlation between (concentration × replication × time) to total variance was strongly trait specific recorded significant mortality (P < 0.05).

**Table 4:** Analysis of variance of the effect of mortality (%) versus concentration, replication and time.

Source	S.S	M.S	df	F	Sig.
C.M	147179.10	2494.56	59	585.09	0.00
I	147679.37	147679.37	1	34637.48	0.00
C	4613.42	659.06	7	154.58	0.00
r	13.97	3.49	4	0.82	0.52
T	52458.16	13114.54	4	3075.95	0.00
C × r	0.00	.	0	.	.
C × T	1381.81	49.35	28	11.58	0.00
r × T	66.33	4.15	16	0.97	0.49
C × r × T	0.00	.	0	.	.

Whereas: S.S (Sum of square); M.S (Mean square); df (Degree of freedom); C.M (Corrected model); I (Intercept); C (Concentration); r (Replication); T (Time); C × r (Concentration × replication); C × T (Concentration × Time); r × T (Replication × Time); C × r × T (Concentration × Replication × Time).

### Probit analysis of ovicidal and larvicidal assays

Probit analysis of data revealed the LC_50_ values, Chi square and fiducial limits at 95% confidence interval (Table 5). In ovicidal assay, the lowest LC_50_ value was demonstrated as 3718.50% at 6 h exposure period followed by 10479.87, 11186.68, 14838.58 and 24001.43 at 72, 12, 48 and 24 h respectively. Whereas, in Larvicidal assay, the lowest LC_50_ value was demonstrated at 72 h being 17.628 exposure period followed by 28.35, 50.49, 215.71 and 482.60 at 48, 24, 12 and 6 h respectively. In larvicidal assay, the LC_50_ value decreases with exposure period. Under *in vitro* conditions, *P. chrysogenum* was showed to be containing more potential in the larvicidal assay as compared to the ovicidal assay.

**Table 5:** Toxicity of Snef1216 (*P. chrysogenum*) against *M. incognita* by ovicidal and larvicidal assay.

Method/Assay used	Time (h)	LC_50_	95% F.L		Slop ± S.E	x^2^
			Lower	Upper		
Ovicidal assay	6	3718.50	1366.24	21827.72	0.57 ± 0.08	2.44
	12	11186.68	1206.68	25620.91	0.35 ± 0.14	19.90
	24	24001.43	2246.52	37389.46	0.26 ± 0.11	14.66
	48	14838.58	2188.46	180229.50	0.27 ± 0.61	1.92
	72	10479.87	1748.35	859768.44	0.28 ± 0.06	6.37
Larvicidal assay	6	482.60	342.74	763.07	1.04 ± 0.08	1.51
	12	215.70	176.90	275.92	1.24 ± 0.08	5.04
	24	50.49	31.54	101.13	0.75 ± 0.12	19.88
	48	28.35	17.76	42.65	1.01 ± 0.13	18.86
	72	17.63	6.09	31.27	1.60 ± 0.30	77.84

### Model validation

Model validation performances for exposure time toward ovicidal and larvicidal assay are listed in Table 6. The exposure time revealed that *R*^2^ values of the ovicidal assay at 6, 12, 24, 48 and 72 h were 0.56, 0.79, 0.88, 0.91 and 0.83 respectively displayed better performances as compared to RMSE. However, exposure time revealed that *R*^2^ values of larvicidal were 0.75, 0.90, 0.97, 0.99 and 0.99 at 6, 12, 24, 48 and 72 h respectively displayed the best curve and best fit to the attained data.

**Table 6:** Regression model for exposure time in response to ovicidal and larvicidal assay.

Exposure time (h)	Ovicidal		Larvicidal	
	*R*^2^	RMSE	*R*^2^	RMSE
6	0.56	2.004	0.75	3.817
12	0.79	5.065	0.91	4.020
24	0.88	7.200	0.97	3.419
48	0.91	7.087	0.99	2.637
72	0.83	10.788	0.99	2.668

Model validation performances for concentration of fermentation of *P. chrysogenum* (Snef 1216) in response to ovicidal and larvicidal assay are listed in (Table 7). The concentration of fermentation revealed that *R*^2^ values of ovicidal at 100%, 75%, 50%, 25%, 10%, 5%, medium and CK were 0.5, 0.5, 0.93, 0.99, 0.99, 0.89, 0.92 and 0.98 respectively displayed better performances as compared to RMSE. However in larvicidal assay the concentrations of fermentation revealed that *R*^2^ values were 0.98, 0.99, 0.98, 0.96, 0.91, 0.85, 0.95 and 0.87 at 100%, 75%, 50%, 25%, 10%, 5%, medium and distill water (control) respectively displayed the best curve and best fit to the attained data.

**Table 7:** Regression model various fermented concentrations of Snef1216 (*P. chrysogenum*) in response to ovicidal and larvicidal assay.

Concentrations	Ovicidal		Larvicidal	
	*R*^2^	RMSE	*R*^2^	RMSE
100%	0.5	1.471	0.98	3.634
75%	0.5	2.093	0.99	2.586
50%	0.93	0.999	0.98	3.204
25%	0.99	0.434	0.96	4.194
10%	0.99	1.123	0.91	4.836
5%	0.89	4.631	0.85	5.761
N. medium	0.92	5.346	0.95	1.190
Distill water	0.98	4.230	0.87	1.121

### Interaction between mortality and hatching

The data presented in (Table 8) revealed the ovicidal and larvicidal activity of *P. chrysogenum* against *M. incognita*. Both mortality and hatching were directly related to the concentration of fermentation and exposure time. Outcomes displayed highly significant (*P* < 0.001) model fitness with (*F* = 888.55, 56845.43, 4022.79, 298.28 and 5053.15) corrected model, intercept, bioassay method, concentration and time. The interaction/correlation between mortality and hatching percentage versus treatment and concentration of fermentation recorded highly significant (P < 0.001) positive correlation with (F = 4831.61), whereas, treatment and exposure time also recorded highly significant (P < 0.001) positive correlation with (F = 334.92). The two-way analysis of variance provides information regarding the interaction between mortality and hatching percentage of *M. incognita* versus concentration and exposure time recorded highly significant results (F = 14.35). Interaction/correlation between (treatment × concentration × time) to total variance was strongly trait specific recorded significant mortality and hatching (P < 0.001 with F = 296.77).

**Table 8:** Analysis of variance of the effect of mortality and hatching (%) versus concentration, replication and time.

Source	S.S	M.S	df	F	Sig.
C.M	254634.39	3223.22	79	888.55	0.00
I	206206.81	206206.81	1	56845.43	0.00
t	14592.64	14592.64	1	4022.78	0.00
C	7574.11	1082.03	7	298.28	0.00
T	73321.27	18330.33	4	5053.15	0.00
t × C	122686.68	17526.67	7	4831.61	0.00
t × T	4859.69	1214.92	4	334.92	0.00
C × T	1457.22	52.04	28	14.35	0.00
t × C× T	30142.80	1076.53	28	296.77	0.00

Whereas: S.S (Sum of square); M.S (Mean square); df (Degree of freedom); C.M (Corrected model); I (Intercept); t (Treatments); C (Concentration); T (Time); t × C (Treatment × Concentration); t × T (Treatment × Time); C × T (Concentration × Time); t × C × T (Treatment × Concentration × Time).

## Discussion

*Meloidogyne incognita* is a dominant root-knot nematode throughout the world. The complete elimination of *M. incognita* from the soil is difficult because of its polyphagous nature. Although different practices are employed to manage this pest however, biological control has become a good alternative to the chemical nematicides. Antagonistic microorganisms are appropriate for controlling nematodes however, still require progressive investigation^12^.

The present study demonstrated that Snef1216 *P. chrysogenum* significantly causes mortality of juveniles and lower the egg hatching of *M. incognita*. However, mortality and hatching are directly related to the concentration of fermentation and exposure time. As mean hatching observed in different concentrations of the medium was found to be similar to that of observed in distilled water. It showed that the medium itself did not show ovistatic or ovicidal property. By the increase in exposure time, there was a correspondingly increased in hatching but all concentrations of fermentation significantly inhibited the hatching rate as compared to control. In our results, the highest ovicidal activities were recorded at 100% concentration. Our findings agreed with Mukhtar and Pervaz^35^ that the medium itself has no ovicidal or ovistatic properties. Hatching inhibition was directly related to the concentration of *Beauveria bassiana*^36^. *Metarhizium anisopliae* was also reported as a biocontrol agent to control the hatching of eggs of *Meloidogyne* spp^37,38^. The culture filtrates of *Aspergillus* sp.*, Trichoderma harzianum*, *T.viride*, *Penicillium*. sp. and *Fusarium*. sp. efficiently control egg hatching of *M. incognita*^39,40^. *Paecilomyces lilacinus* can easily parasitize females, juveniles and eggs of nematodes; therefore, it efficiently reduced their population in soil^41–43^. The conidia and hyphae of fungus can easily be penetrated in the eggs, resulting in larval death within egg^44^. Many fungal strains are capable to control reproduction and development of root knot nematodes in different crops^25,45^.

Our results also revealed that all concentrations of fermentation possess the nematicidal effect of varying degree on *M. incognita*. The potential of Snef1216 was the maximum, with J2 mortality up to 97.8% at 72 h at 100% concentration. However, their mortality response was concentration and time exposure dependent. Gapasin, *et al*.^46^ and Pau, *et al*.^47^ demonstrated that *P. lilacinus* culture filtrate had 100% nematicidal effect on J2 of *Meloidogyne spp*. Our results also consistent with Migunova, *et al*.^28^ that mortality percentage is directly related to the fermentation’s concentration and the exposure time. *Aspergillus* sp., *Fusarium* sp., *Penicillum* sp., *T. harzianum* and *T. viride*, were showed nematicidal properties on juveniles of *M. incognita* and exhibited more than half of juveniles mortality during 24 h incubation at 25% concentration of these fungal culture filtrate^39^. Hussain, *et al*.^48^ demonstrated that the effect of *T. harzianum* species on inactivation of J2 of *Meloidogyne hapla* reached 70.2% after 48 hours and 86% after 72 hours of its incubation in *Clonostachys rosea* strain. Regaieg, *et al*.^49^ reported that mortality of juveniles was relative to the *Verticillium leptobactrum* filtrate concentrations and the duration of exposure. Similarly Uddin, *et al*.^18^ reported that the culture filtrate of fungus *P. chlamydosporia* was effected to control *M. incognita*. The practice of biocontrol agents may decrease the use of harmful chemicals. However, their efficacy mainly depends upon species of nematode^50,51^. The outcomes of the present study clearly demonstrated that the use of Snef1216 (*P. chrysogenum*) for the controlling of *M. incognita* is effective and eco-friendly as compared to chemical nematicides.

## Conclusions

Controlling of *M. incognita* by using the antagonistic fungus is a safe, effective and eco-friendly method as compared to injurious synthetic chemical nematicides. Snef1216 (*P. chrysogenum*) is apparently effective and environmentally friendly nematicides which enhanced mortality with increasing concentration of fermentation and exposure time. It is concluded that *M. incognita* has reasonable sensitivity toward Snef1216 (*P. chrysogenum*). It is reasonable to suppose that quick mortality and hatching inhibition effects connected with toxic metabolites released by the tested strain. The compounds of these metabolites and mechanisms of their actions need to be further investigations.

## Materials and methods

### Collection and activation of Strain Snef1216 (Penicillium chrysogenum)

Strain Snef1216 (*P. chrysogenum*) was obtained from China General Microbiological Culture Collection Center and stored at −80 °C in Nematology Institute of Northern China^52^. A small quantity of strain Snef1216 (*P. chrysogenum*) was put into PDA filled cavities and then placed in an incubator at 25-28 °C for 7 days to check the purity and activation. A pure single colony of strain was selected for further procedure (Fig. 1).

### Fermentation

For the preparation of nutrition medium 0.003 g FeSO_4_.7H_2_O, 0.08 g MgSO_4_.7H_2_O, 0.4 g KCl, 2 g K_2_HPO_4,_ 8 g NaNO_3_ and 50 g sucrose were added into 1 L of distilled water and then the resulting mixture was boiled for 3-5 minutes. After that, 100 ml nutrition medium was poured into the 250 ml conical flasks and sterilized in the steam autoclave machine at 121 °C for 30 min. Single pure fungus colony was cut into small pieces about 1 mm in diameters by the sterilized cutter. About five pieces were added into 100 ml of sterilized nutrition medium and placed in a shaker at 28 °C, at 150 rpm for 3 days. After that 100 ml new medium was poured into each flask and put again in a shaker for 8 days at 28 °C and150 rpm and filtered and stored at 4 °C^52^.

### Nematode Inoculums

The population of *M. incognita* was cultured in tobacco plants grown in the greenhouse of Nematology Institute of Northern China (NINC), Shenyang Agricultural University (SYAU), Shenyang, Liaoning, China. Plants were carefully plucked and roots were chopped into 1-2 cm slices and then macerated in a small amount of water by using the electric blender. 0.05% sodium hypochlorite (NaOCl) was added into macerate. The mixture was manually shaken for 1-2 minutes to separate the eggs from the gelatinous matrix. Then, the suspension was poured through 200 and 500 sieve meshes respectively and washed with tap water to remove NaOCl. Eggs were further purified by centrifugation in 454gL^−1^ sucrose for 4 minutes at 3000 rpm. The supernatant was poured into 500 sieve mesh and rinsed several times with sterilized water. Eggs inoculums were transferred into a funnel and allowed to hatch into second stage juveniles (J2s). These J2s were then allowed to crawl through eight layers of Kim-wipe tissues into sterilized water by using the Baermann funnel method^53^. The J2s in the resulting suspension was adjusted at 100 J2/0.5 ml and also eggs suspension adjusted at 100 eggs/0.5 ml for subsequent experiments.

### *In vitro* ovicidal assay

Ovicidal efficacy was evaluated by slight modification in process of Su and Mulla^54^. One hundred eggs of *M. incognita* were shifted into all of 24-well microliter plates containing different concentrations of fermentation (100%, 75%, 50%, 25%, 10% and 5%), nutritious medium and distilled water separately. The microliter plate was covered and incubated at 28 °C under humidified conditions for 72 h. The hatching of eggs was microscopically monitored after 6, 12, 24, 48 and 72 h exposure time. After 72 h, Lugol’s iodine solution (300μL) was put into all wells to stop more hatching of eggs^55^. To ascertain whether Snef1216 (*P. chrysogenum*) had ovistatic or ovicidal property the unhatched eggs were transferred to distilled water to observe hatching after exposure^56,57^. Five replicates were analyzed for all treatments for the accurateness of results. The percentage of hatching was calculated by the formula^13^.

$${\rm{Hatching}}=\frac{{\rm{number}}\,{\rm{of}}\,{\rm{hatched}}\,{\rm{eggs}}}{{\rm{total}}\,{\rm{number}}\,{\rm{of}}\,{\rm{eggs}}}\times 100$$

### *In vitro* larvicidal assay

One hundred second-stage juveniles of *M. incognita* carefully shifted into each well of 24-well microliter plate containing different concentrations of fermentation (100%, 75%, 50%, 25%, 10% and 5%), nutritious medium and distilled water separately. The microliter plate was incubated at 28 °C under humidified conditions for 72 h. J2’s populations were microscopically monitored after 6, 12, 24, 48 and 72 h exposure period. To ascertain whether Snef1216 (*P. chrysogenum*) had larvistatic or larvicidal property the immotile J2s were washed and transferred into distilled water to observe resumption of motility^56,57^. Juveniles were considered as dead when they remained immotile on probing with fine hair-needle and percentage mortality was calculated^55^. Five replicates were analyzed for all treatments for the accurateness of results.

$${\rm{Mortality}}\,=\,\frac{{\rm{number}}\,{\rm{of}}\,{\rm{dead}}\,{\rm{juveniles}}}{{\rm{total}}\,{\rm{number}}\,{\rm{of}}\,{\rm{juveniles}}}\times 100$$

### Model validations

The association was used to construct a model for concentrations and exposure periods and the evaluation of the coefficient of determination (*R*^2^) values with root mean square error (RMSE). RMSE and *R*^2^ denote the standard deviation of the estimated errors (residuals). Prediction errors express how many values are far away from and around the regression lines. The performance of the model in the concentrations and exposure periods were assessed by RMSE. RMSE was calculated by the Equation of Debaeke, *et al*.^58^.

$$RMSE\,=\,1/n\sqrt{{\sum }_{i=1}^{n}{({X}_{obs,i}-{X}_{model,i})}^{2}}$$

### Data analysis

Ovicidal and larvicidal data were analyzed by two-way analysis of variance for calculating the interaction between ovicidal and larvicidal assay, concentrations and post-treatment time (exposure period) while mean between treatments were calculated for significance test by Duncan’s multiple range test at *P= 0.05*. All statistical processes were administered by different statistical packages such as EPA Probit analysis program (version 1.5) software, IBM-SPSS statistics (version 25.0) software and MS Excel.
